# Wetscapes: Restoring and maintaining peatland landscapes for sustainable futures

**DOI:** 10.1007/s13280-023-01875-8

**Published:** 2023-05-24

**Authors:** Ralph J. M. Temmink, Bjorn J. M. Robroek, Gijs van Dijk, Adam H. W. Koks, Sannimari A. Käärmelahti, Alexandra Barthelmes, Martin J. Wassen, Rafael Ziegler, Magdalena N. Steele, Wim Giesen, Hans Joosten, Christian Fritz, Leon P. M. Lamers, Alfons J. P. Smolders

**Affiliations:** 1 Environmental Sciences, Copernicus Institute of Sustainable Development, Utrecht University, Princetonlaan 8a, 3584 CB Utrecht, The Netherlands; 2 Aquatic Ecology and Environmental Biology, Radboud Institute for Biological and Environmental Sciences, Radboud University, Heyendaalseweg 135, 6525 AJ Nijmegen, The Netherlands; 3 School of Biological Science, University of Southampton, Southampton, SO17 1BJ UK; 4 B-WARE Research Centre, Toernooiveld 1, 6525 ED Nijmegen, The Netherlands; 5 Institute of Botany and Landscape Ecology, University of Greifswald, Partner in the Greifswald Mire Centre, Soldmannstr. 15, 17487 Greifswald, Germany; 6 Department of Management, HEC Montréal, Édifice Côte-Sainte-Catherine 3000, Chemin de La Côte-Sainte-Catherine, Montreal, Canada; 7 Associate with Naturalis Biodiversity Center, Darwinweg 2, 2333 CR Leiden, The Netherlands

**Keywords:** Carbon, Paludiculture, Restoration, Rewetting, Sustainable peatland use, Wetland

## Abstract

Peatlands are among the world’s most carbon-dense ecosystems and hotspots of carbon storage. Although peatland drainage causes strong carbon emissions, land subsidence, fires and biodiversity loss, drainage-based agriculture and forestry on peatland is still expanding on a global scale. To maintain and restore their vital carbon sequestration and storage function and to reach the goals of the Paris Agreement, rewetting and restoration of all drained and degraded peatlands is urgently required. However, socio-economic conditions and hydrological constraints hitherto prevent rewetting and restoration on large scale, which calls for rethinking landscape use. We here argue that creating integrated *wetscapes* (wet peatland landscapes), including nature preserve cores, buffer zones and paludiculture areas (for wet productive land use), will enable sustainable and complementary land-use functions on the landscape level. As such, transforming landscapes into *wetscapes* presents an inevitable, novel, ecologically and socio-economically sound alternative for drainage-based peatland use.

## Peatland degradation by drainage

Peatlands are terrestrial ecosystems that as a result of wet and anoxic conditions have accumulated a large amount of organic matter over decadal to millennial timescales (Yu et al. 2010). Moreover, peatlands cover only 3% of the land surface, yet store over 30% of the world’s soil organic carbon (Gorham 1991; Leifeld and Menichetti 2018; Xu et al. 2018). Carbon densities of on average 1500 Mg C ha^−1^ make peatlands unrivalled carbon stores, largely exceeding densities of 200 and 900 Mg C ha^−1^ as reported for forests and mangroves, respectively (Temmink et al. 2022). Furthermore, peatland ecosystems play an important role in nutrient storage and cycling, freshwater purification and retention, and maintaining unique biodiversity (Zedler and Kercher 2005; Jurasinski et al. 2020).

Peatlands have been drained on a large scale mainly for agriculture, forestry and peat extraction (Joosten and Clarke 2002; Fluet-Chouinard et al. 2023). Drainage-based peatland exploitation flourished for centuries in Northern countries. Over the last decades, this land-use type gained traction in Southeast Asia (Rawlins and Morris 2010) and still expands despite mounting evidence that it is unsustainable (Evers et al. 2017; Pelsma et al. 2020). Perceived economic benefits hitherto did not consider the societal costs of drainage-associated greenhouse gas (GHG) emissions, nutrient leaching, land subsidence, loss of water purification and retention capacity, and loss of biodiversity (Hutchinson 1980; Hooijer et al. 2012; Turetsky et al. 2015; Günther et al. 2020). Large areas of peatlands were entirely lost and 12% of all remaining peatlands worldwide are degraded due to human activities (Leifeld and Menichetti 2018; Günther et al. 2020; UNEP 2022). Most peatland loss and degradation has taken place in Europe, Southeast Asia and China (Leifeld and Menichetti 2018; UNEP 2022). In Europe, 270,000 km^2^ of peatlands were drained over the last centuries, with 54% remaining more or less intact (UNEP 2022). Even more dramatic destruction took place in Southeast Asia with the drainage and deforestation of 71% of Malaysian and Western Indonesian peat swamp forests since the 1990s (133,000 km^2^), and only 6.4% remaining in more or less pristine condition (Miettinen et al. 2016). Furthermore, drainage makes peatlands more susceptible to peat fires, which are expected to increase under climate change (Kettridge et al. 2015; Page and Hooijer 2016). As an example, the 1997 peat fires in Indonesia impacted 730,000 ha, emitted 0.19–0.23 Gt carbon through peat combustion resulting in 25–85 cm of peat loss, and negatively affected the health of local inhabitants (Page et al. 2002; Kiely et al. 2021). The total national damage costs of these events have been estimated at tens of billions of euros (Gaveau et al. 2021; Kiely et al. 2021).

Drained and degraded peatlands cover only 0.3% of the world’s land surface (i.e., 12% of all current peatlands), but emit 4% of the total human-induced global greenhouse gas (GHG) emissions and even more when including fires (Leifeld and Menichetti 2018; Friedlingstein et al. 2020; UNEP 2022). Agricultural crops on deeply drained peatland contribute disproportionately to these numbers; the 1.1% of all crops produced on drained peatlands account for 32% of the total cropland GHG emissions worldwide (Carlson et al. 2017). In addition, peatland drainage leads to land subsidence with rates ranging from 0.5 to 20 mm year^−1^ in temperate regions (Lipka et al. 2017; Ikkala et al. 2021) and up to 50 mm year^−1^ in the tropics (Hooijer et al. 2012; Giesen and Sari 2018). In the Netherlands, home to a human population of *c.* 17 million, estimated costs related to land subsidence caused by peat oxidation range from 1.7 to 5.2 billion euros from 2010 to 2050 (van den Born et al. 2016). Costs of renovating foundations of buildings on peat soils will over that period add an extra 5–38 billion euros (van den Born et al. 2016) or even as much as 80 billion euros (Dutch Knowledge Center Approach to Foundation Problems). Moreover, land subsidence combined with rising sea levels increases flood risk and salt water intrusion in coastal zones (Barlow and Reichard 2010; Herbert et al. 2015). With sea levels expected to rise (Dayan et al. 2021; Masson-Delmotte et al. 2021), further subsidence will cause many drained coastal peatlands to be flooded and degraded due to salinization (Herbert et al. 2015; Hooijer et al. 2015; van Dijk et al. 2015), ultimately leading to a substantial loss of productive land.

Responses for peatland restoration to mitigate the negative environmental effects of current peatland use emerge across the globe, but these efforts are very often local scale focussed and hardly enough to reach the restoration goals. Considering the climate and biodiversity crises, an ambitious integrated *wetscape* approach is needed that allows rewetting of all drained peatlands worldwide and focuses on long-term sustainability at the landscape scale.

## Creation of sustainable *wetscapes*

**Fig. 1:**
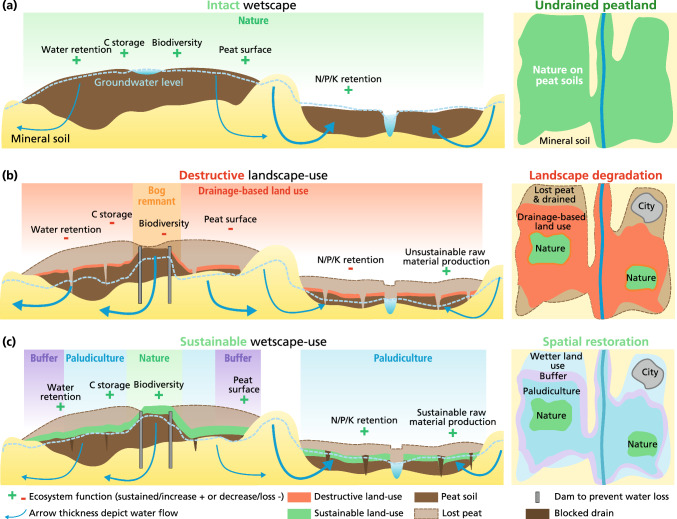
**Intact wetscape, destructive landscape-use, and sustainable wetscape-use**. (**a**) Conceptual representation of an intact *wetscape* – a *wetscape* does not always consists of the depicted features – that consists of a raised bog (left), dry mineral soils (middle), and a groundwater-fed fen (right). Natural peatlands retain water, store carbon and nutrients and host characteristic species. (**b**) Destructive land-use, including drainage-based agricultural and forestry use and peat extraction, which results in land subsidence, carbon losses through peat oxidation, reduced water holding capacity, nutrient release, all affecting biodiversity. Landscape-wide drainage influences near-natural peatlands, resulting in lateral and vertical water losses, despite human interventions to prevent water losses (e.g., dam construction). (**c**) Creation of a sustainable *wetscape*: protected core (near) natural peatland, surrounded by wet agriculture (paludiculture), fringed by buffer zones with lower natural values. Rewetting aims to restore ecosystem services and processes, such as carbon storage and water and nutrient retention, and to prevent land surface subsidence (and possibly even re-installs a gain in surface height), while allowing sustainable production of raw materials

Landscape-scale peatland restoration, to be successful and adopted widely, should embrace the different—sometimes opposing—societal interests (e.g., nature conservation versus agricultural production) and acknowledge the wide variety of stakeholders. The mitigation of negative environmental impacts requires full rewetting of all drained peatlands (Günther et al. 2020; Jurasinski et al. 2020; Convention on Wetlands 2021; UNEP 2022; Hiller and Fisher 2023). This aim is, however, frustrated by the trillions of euros/dollars that have been invested in drainage infrastructure to support agricultural land-use and the concomitant cultivated perception that draining peatlands is good practice. The necessary complete rewetting can, therefore, only be achieved by creating *wetscapes*, wet peatland landscapes, that combine a variety of functions and management options in a sustainable and integrated, mutually reinforcing spatial setting (Fig. 1):

(i) *core areas of conserved or restored near-natural peatlands* with the aim to preserve and re-install unique natural biodiversity and high carbon sequestration and storage (‘wet wilderness’), which are fringed by
(ii) *rewetted peatlands utilized for the production of biomass* in a way that preserves the peat body and minimizes greenhouse gas emissions, i.e., paludiculture (wet agriculture and forestry) and are embedded in
(iii) *rewetted peatlands that function as hydrological and hydrochemical buffer zones against negative impacts of adjacent intensive land use on mineral soils.*

**Fig. 2:**
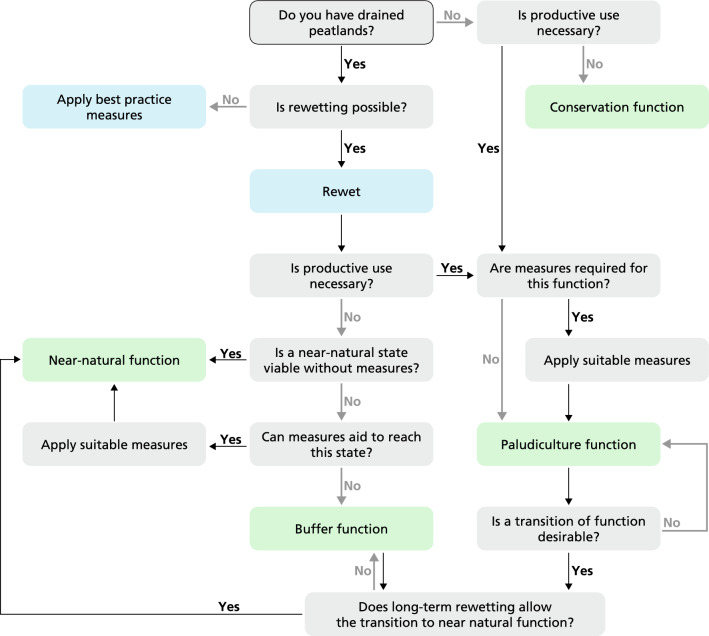
**A decision tree with land-use change pathways for wetscape creation**. The decision tree leads to four functions in green (conservation, near-natural, buffer and paludiculture function), important measures in blue (rewetting and best practises in the case that rewetting is impossible) and the transition over time and space between functions

Overall, rewetting and restoring peat accumulation will provide emerging benefits by offsetting methane production by mitigating carbon emission and enhancing sequestration (Günther et al. 2020; Mrotzek et al. 2020), freshwater water retention and flood control, and nutrient storage and removal (Bonn et al. 2016; Vroom et al. 2020). Finally, we envision that these functions may transition over space and time (Fig. 2).

### Core reserves: Peatland conservation and restoration

The conservation and restoration of core peatland reserves aim to preserve biodiversity and sustain important ecosystem services. Such natural areas are under pressure by adjacent land-use, including drainage, (over)exploitation of resources and atmospheric nitrogen deposition (Lamers et al. 2015; UNEP 2022; Hiller and Fisher 2023). Successful peatland conservation and restoration starts by keeping wet areas wet and making formerly drained peatlands wet again (rewetting) (Minayeva et al. 2017; Renou-Wilson et al. 2019; Convention on Wetlands 2021). Especially peatlands that have only been subject to drainage and fertilization to a limited extent have high potential to transition to core reserves (Convention on Wetlands 2021) (Fig. 1).

### Paludiculture: Transforming agricultural landscapes

Paludiculture—wet productive peatland use –in a *wetscape* may act in concert with outer buffer zones to protect the core peatland reserves by keeping them wet and to prevent negative influences from the surrounding non-*wetscape* area. Additionally, paludiculture offers an alternative business model for current environmentally harmful drainage-based peatland use (Fig. 1).

Paludiculture may produce biomass for construction, fuel, fodder, growing media, food or medicine (Wichtmann et al. 2016; Ziegler et al. 2021) (Fig. 1). The question of which crops to farm and which raw materials to produce depends on site conditions, climate and management (Wichtmann et al. 2016; Geurts et al. 2019). Paludiculture in temperate fens currently focuses on growing plants for insulation and fodder (*Typha*), thatch (*Phragmites*), wood/timber (*Alnus*), direct energy/heat generation or biogas production (*Typha*, *Phragmites*, *Carex*, *Phalaris* etc.) and food (e.g., wild rice and berries) (Wichtmann et al. 2016; Geurts et al. 2019) but much more options do exist (Abel and Kallweit 2022). In temperate bogs, paludiculture focuses on growing peatmoss (*Sphagnum*) biomass as a renewable raw material for horticultural growing media to replace fossil *Sphagnum* peat (Gaudig et al. 2018). In the tropics, traditional paludiculture focuses on Sago (*Metroxylon sagu*) for starch or Illipe Nut (*Shorea stenoptera*) as cocoa butter substitute (Joosten et al. 2012; Abel et al. 2013), though a much wider range of products are being trialled (Giesen 2021). Paludiculture also offers opportunities for wet animal husbandry, such as water buffalo for meat and dairy (Sweers et al. 2014), or freshwater fish in Southeast Asia (Setiadi and Limin 2015). Paludiculture may be best established in locations where complete restoration is challenging and a well-developed infrastructure for production (e.g., water management, product chains, markets) exists.

Paludicultures can facilitate high yields by optimizing site conditions and selecting optimal crops (Gaudig et al. 2018). Precise water table regulation allows the optimization of carbon sequestration and storage. An average annual water level of 10 cm below the peat surface maximizes peat formation and carbon dioxide (CO_2_) uptake and limits methane (CH_4_) emissions, which may occur at higher water levels (Couwenberg et al. 2011; Evans et al. 2021). In addition, high water levels prevent peat fires (Putra et al. 2018). Management of (particularly eutrophic) irrigation ditches needs special attention as these may be notorious hotspots of CH_4_ emissions (Schrier-Uijl et al. 2010; Peacock et al. 2021).

Paludiculture may provide multiple ecosystem services, which may change over time. For example, while *Sphagnum* paludiculture focuses on producing renewable resources for growing media, the rewetted production site prevents CO_2_ emissions (Günther et al. 2017), supports biodiversity (Muster et al. 2020), and removes nutrients (Temmink et al. 2017; Vroom et al. 2020). Furthermore, the harvesting of plants removes the nutrients sequestered in the biomass (Zerbe et al. 2013; Geurts et al. 2020). As such, paludiculture may gradually lower nutrient availability and facilitate the transition from nutrient-rich and highly productive paludiculture to less nutrient-demanding but higher quality crops or even on the long-term to nutrient poor and biodiverse near-natural peat-forming ecosystem (Smolders et al. 2008; Jabłońska et al. 2021) (Fig. 2). The latter developments need to take the economic transition dynamic into account, such as decreasing yields, changing paludiculture crops, or even discontinuing paludicultural practise.

### Buffer zones: Rewetted peatlands as hydrological buffers

Drainage alters the hydrological functioning of entire landscapes, and negatively impacts biodiversity and ecosystem services of (near)-natural peatlands and paludicultures even without direct water diversion and extraction (Holden et al. 2006; Yule 2010; Krejčová et al. 2021). As such, rewetted (peat)land surrounding core peatland reserves and paludicultures can function as a hydrological buffer against adjacent high-intensity land use (Fig. 1). Ideally, rewetted buffer zone peatlands will allow the rise and stabilization of the water table in core peatland reserves and paludicultures, while not negatively affecting (but even enhancing) agricultural productivity on adjacent mineral soils (Joosten et al. 2015; Ahmad et al. 2020).

The additional values of wetter landscapes include lowering land surface temperatures and pollution control (Wu et al. 2021). For example, channelling nutrient-enriched surface water through the wet buffer (peat)land will lead to uptake of nutrients by vegetation (i.e., phytoremediation) and soil and to denitrification, lowering nutrient input in the core peatland reserve (Adler et al. 2008; Cusell et al. 2014; Vroom et al. 2018) and may also lower water hardness and sulphate concentrations (Lamers et al. 2015; Van Diggelen et al. 2020). Care has to be taken, however, that the internal mobilization of nutrients in the formerly fertilized, rewetted peatland does not lead to eutrophication of the core reserve (Smolders et al. 2006; Van Diggelen et al. 2020).

The buffer zone is not fixed in time and in the best case will transition to a core peatland reserve (Fig. 2). Measures to improve site conditions to guide the transition of degraded towards biodiverse peatlands may involve nutrient attenuation, topsoil removal and species introduction (Smolders et al. 2008; Emsens et al. 2015; Van Diggelen et al. 2020; Convention on Wetlands 2021; Quadra et al. 2023).

## The potential of *wetscapes*

**Fig. 3:**
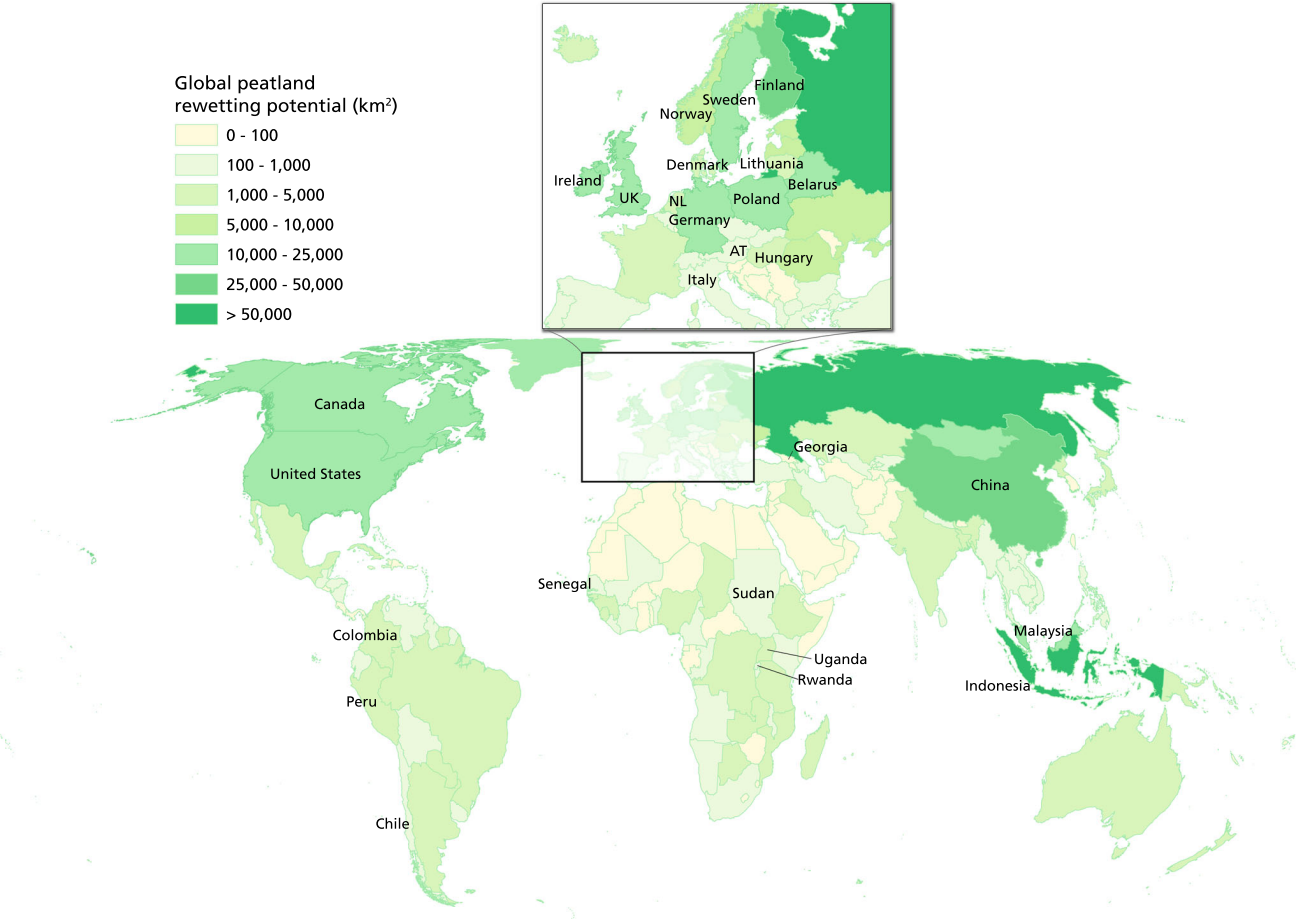
**Global peatland rewetting potential and countries with paludiculture pilots**. Colours illustrate the estimated potential for peatland rewetting per country (km2), which is based on the extent of degraded peatland area (source: Greifswald Mire Centre – Global Peatland Database). Country names indicate where paludiculture pilots are ongoing with an inset of Europe for detail (after (Geurts et al. 2019; Ziegler et al. 2021))

To reach global climate goals, the implementation of *wetscapes* should take place on 570,000 km^2^ (57 million ha) of peatlands that are degrading worldwide (UNEP 2022) (Fig. 3). With respectively 272,000 (27.2 million ha) and 209,000 km^2^ (20.9 million ha) of degraded peatlands (UNEP 2022), Europe and Southeast Asia emerge as global transformation hotspots. To successfully transform drainage-based landscapes into *wetscapes* we advise a step-wise implementation of the spatial setting (nature, buffer, and paludiculture). This concept is based on (i) small (ha) to larger (thousands of ha)-scale pilot projects with intensive and cross-disciplinary monitoring to gather sound scientific evidence, that (ii) are part of a large-scale and longer-term (10–30 years) innovation and transformation strategy, which (iii) accounts for costs to consumers and producers who have to change lifestyles, and that (iv) involves a policy mix that encourages new practices and prevents and terminates drainage-based peatland use (Mazzucato 2018; Ziegler 2020). Wider implementation of the *wetscape* approach needs to account for several prerequisites. *Wetscapes* demand ample water to prevent peat desiccation during summer (Page et al. 2002; Thompson and Waddington 2013). In many countries, water infrastructure is designed for fast water discharge, which frustrates water-use efficiency and calls for the restoration of landscape hydrology over complete catchments. As the presence of peat indicates a local (former) water surplus, retaining, preserving, and re-using water, instead of discharging it into the sea as fast as possible, will strongly improve the perspectives of large-scale peatland rewetting. Next to water quantity, water quality—which varies between and within countries—determines which function aligns with a specific rewetted peatland. However, one should note that surface water and groundwater quality will improve over time as a result of rewetting and new peat formation (Van Diggelen et al. 2020; Vroom et al. 2020). The feasibility of creating a *wetscapes* is thus context dependent and strongly depends on landscape morphology, peatland type and local eco-(hydro-)logical setting (Joosten et al. 2017).

Beyond landscape eco(hydro-)logical issues, challenges include selecting and testing prospective crops, improving their cultivation and harvesting techniques, and developing value chains, industries and markets for the novel products that emerge from paludiculture (Wichtmann and Joosten 2007; Giesen 2021). In the near future, *wetscape* farmers should have a portfolio of crops that provide a secure income, while industries need a constant influx of high-quality raw materials for production and sales. Nowadays, farmers still receive subsidies for climate-damaging agriculture on drained peat soils, whereas paludiculture often remains ineligible. Large-scale implementation requires mainstreaming paludiculture by long-term support and income guarantees to raise trust in future economic viability and to ‘level the playing field’.

The public perception of rewetting may hinder rapid large-scale realization of *wetscapes*. Previous generations have painstakingly reclaimed wet ‘wastelands’ to turn them into ‘valuable’ and ‘productive’ fields, pastures and forests and the current generation views the drained peatland landscape as home and a source of identity (Wichtmann et al. 2016; Ziegler 2020; Flood et al. 2021). Consequently, the idea to turn back these lands into wetlands could invoke substantial opposition. Yet, novel narratives on meaningful and responsible land-use and changing socio-economic perspectives (e.g., paludiculture) will most likely rapidly increase social acceptance. In the Netherlands, famous for its technological water management, the traditional attitude has—after some serious flood events in the 1990s—slowly shifted from fighting against towards moving along with water and adopting landscape-scale measures. More recently, the ever more prominently visible downsides of peatland drainage have led to an attitude in which freshwater qualifies as a scarce resource that warrants sustainable management (Rijksoverheid 2021). Moreover, a discrete-choice experiment with Danish, German, and Polish citizens towards ecosystem services of the Baltic Sea basin pointed to a willingness to pay substantial amounts for restoration measures including wild wetlands and wetland agriculture (Giergiczny et al. 2022).

## Conclusions

Human-induced climate change and biodiversity loss forces humanity to drastically reduce GHG emissions and to restore ecosystems on a global scale. Re-creating and maintaining peatland-dominated *wetscapes* as an alternative for drainage-based land use provides benefits for both nature and human societies and is socio-economically feasible. The implementation of *wetscapes,* rather than just restoring and conserving peatland cores, leads to the recovery of biodiversity, water retention, carbon sequestration, cooling of land surface, and great reduction of nutrient emissions. Most importantly it will substantially reduce greenhouse gas emissions and allow farmers to produce crops sustainably and act again as real stewards of the land.
