# Correction: Wetscapes: Restoring and maintaining peatland landscapes for sustainable futures

**DOI:** 10.1007/s13280-023-01890-9

**Published:** 2023-06-23

**Authors:** Ralph J. M. Temmink, Bjorn J. M. Robroek, Gijs van Dijk, Adam H. W. Koks, Sannimari A. Käärmelahti, Alexandra Barthelmes, Martin J. Wassen, Rafael Ziegler, Magdalena N. Steele, Wim Giesen, Hans Joosten, Christian Fritz, Leon P. M. Lamers, Alfons J. P. Smolders

**Affiliations:** 1 Environmental Sciences, Copernicus Institute of Sustainable Development, Utrecht University, Princetonlaan 8a, 3584 CB Utrecht, The Netherlands; 2 Aquatic Ecology and Environmental Biology, Radboud Institute for Biological and Environmental Sciences, Radboud University, Heyendaalseweg 135, 6525 AJ Nijmegen, The Netherlands; 3 School of Biological Science, University of Southampton, Southampton, SO17 1BJ UK; 4 B-WARE Research Centre, Toernooiveld 1, 6525 ED Nijmegen, The Netherlands; 5 Institute of Botany and Landscape Ecology, University of Greifswald, Partner in the Greifswald Mire Centre, Soldmannstr. 15, 17487 Greifswald, Germany; 6 Department of Management, HEC Montréal, Édifice Côte-Sainte-Catherine 3000, Chemin de La Côte-Sainte-Catherine, Montreal, Canada; 7 Associate with Naturalis Biodiversity Center, Darwinweg 2, 2333 CR Leiden, The Netherlands

**Correction to: Ambio** 10.1007/s13280-023-01875-8

In the original publication, an arrow was missed in the left side of Fig. 2. The correct version of Fig. 2 is provided in this correction.

The original article has been corrected.

**Fig. 2:**
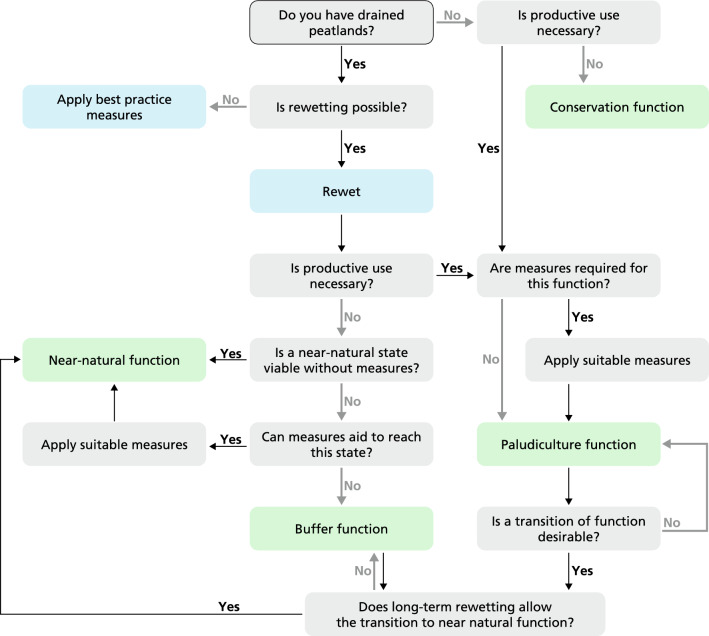
**A decision tree with land-use change pathways for wetscape creation**. The decision tree leads to four functions in green (conservation, near-natural, buffer and paludiculture function), important measures in blue (rewetting and best practises in the case that rewetting is impossible) and the transition over time and space between functions

